# Treatment of Bleeding Diathesis Associated with a Heparin-Like Anticoagulant in Plasma Cell Neoplasia Using Protamine

**DOI:** 10.1155/2018/4342301

**Published:** 2018-06-13

**Authors:** Christopher A. Willner, Mohammed M. Chisti

**Affiliations:** ^1^Department of Internal Medicine, Beaumont Royal Oak, Royal Oak, MI, USA; ^2^Department of Hematology and Oncology, Beaumont Royal Oak, Royal Oak, MI, USA

## Abstract

The development of a heparin-like anticoagulant (HLAC) in plasma cell neoplasia has previously been described in clinical literature. Testing of this HLAC, primarily in vitro, has demonstrated that neutralization may be achieved with protamine sulfate, owing to antithrombin III cofactor activity. We report a case in which intravenous protamine sulfate was administered to a patient with IgG-kappa monotypic multiple myeloma, which resulted in resolution of bleeding and coagulopathy, confirmed via objective laboratory data. Our case is intended to demonstrate that intravenous protamine sulfate should be considered in acute bleeding with plasma cell neoplasia. We review the literature to observe past experiences about this phenomenon. We postulate that chemotherapy, targeted therapies, and immunotherapies may also disrupt production of a HLAC. With further investigation, this strategy could be applicable in other hematological malignancies with bleeding diathesis, chiefly if the pathophysiology of the HLAC is precisely defined.

## 1. Introduction

Bleeding is a challenging area of caring for patients with multiple myeloma, and while several mechanisms have been proposed, it remains incompletely understood. Heparin-like anticoagulants (HLAC) have been studied as a mechanism by which bleeding occurs [1], and owing to the mechanism of action, protamine has been proposed as a potential therapy [2]. More recent translational data have demonstrated that this HLAC may be the paraproteins produced within myeloma [2]. Other analyses have concluded that proteins similar to heparin sulfate may be responsible by binding antithrombin and leading to activation of the heparin binding site, but their origin not clearly elucidated [3].

## 2. Case Presentation

A 62-year-old Columbian female was diagnosed in 2008 with multiple myeloma (MM). Flow cytometry revealed an IgG kappa monotypic plasma cell population, expressing CD33, CD38, CD45, CD56, CD117, CD138, and kappa light chains. The plasma cells were CD19 negative. IgG was measured at 6390 mg/dL, and immunoglobulins of all other types were decreased. Hematopathology revealed extensive bone marrow involvement (90%) by plasma cells, nearly absent iron stores, moderate normocytic normochromic anemia, reticulocytopenia with prominent rouleaux formation, moderate thrombocytopenia, and absolute lymphopenia. Cytogenetics revealed an abnormal hyperdiploid karyotype including a der(19)*t*(1q;1p) chromosome with additional copies of CCND1, RB1, and LAMP1, loss of chromosome 17 centromere, and p53 gene disomy. The patient was first started on lenalidomide and dexamethasone and progressed despite multiple chemotherapeutic regimens including bortezomib and dexamethasone, additional cycles of lenalidomide and dexamethasone, melphalan with thalidomide and prednisone (MPT), and bortezomib and dexamethasone. The patient reported complete adherence to treatment.

Throughout the course of treatment, the patient was admitted several times with right flank pain, hematuria, and persistent hypercalcemia. The patient also had a notable past medical history of CKD secondary to myeloma kidney, and asthma.

The patient was admitted for severe hematuria and epistaxis in February 2011. The patient was also hypercalcemic and hyperkalemic and received both zoledronic acid and kayexalate. At that time, the patient underwent an extensive coagulation profile including screening for lupus anticoagulant, antiphospholipid syndrome, paraproteins, and factor levels. Ristocetin cofactor assay revealed a level of 46% (normal 50%–150%), indicating the absence of a Von Willebrand Factor deficiency. Von Willebrand Factor Antigen was >300 IU/dL (normal 60–150 IU/dL). Notably, thrombin time (TT) was elevated to 32.3 sec (normal 15–19 sec). The etiologies considered included increased fibrin split products, dysfibrinogenemia or a deficiency of fibrinogen, heparin treatment, and heparin-like molecules, to name a few. Fibrinogen was 228 mg/dL (normal 175–400 mg/dL), and fibrin split products were <10 mcg/mL (normal <10 mcg/mL), which also suggested the presence of another inhibitor. Factor Xa screen was 0.1 U/mL (normal 0.3–0.7 U/mL), which indicated the absence of UFH and LMWH leading to coagulopathy. Abnormalities of fibrinogen were also ruled out with a normal reptilase time of 22 sec, and control measured during reptilase time testing was 21 sec. PT was 12.0 sec (normal 9.6–11.5 sec), and INR was 1.1 (normal < 1.2). A mixing study was conducted to investigate for the presence of inhibitors; PTT (patient) was 44.3 sec (normal 25–32 sec) and was only partially corrected with mixing; PTT of control plasma was 27.8 sec, PTT Mix 1 : 1 control and patient was 34.2 sec, PTT control following 2 hour incubation was 29.4 sec, and PTT Mix 1 : 1 with 2 hour incubation was 36.5 sec (25–32 sec). Notably also, factor VIII activity was elevated at 273% (normal 45–150%), which indicated an increased risk for venous thrombosis. Continuous protamine sulfate IV of 2–7 mg/hour was given, which led to notable improvement and subsequent resolution of our patients' epistaxis and hematuria. A hematoma formed after a bone marrow biopsy was performed on the same admission, for which surgical consultation was requested. No intervention was done, and resolution of the hematoma occurred spontaneously.

## 3. Discussion

Clinical data have suggested the presence of an acquired heparin-like coagulation inhibitor in plasma cell neoplasia that is responsible for bleeding diathesis [1–5]. Characteristic features previously reported include a prolonged plasma thrombin time with laboratory evidence of an inhibitor as well as a near-normal reptilase time [6]. Additionally, it has been reported that following mixing study, reptilase time does not correct, and as expected, there is incomplete correction of prothrombin time [7]. An analysis of a similar clinical scenario by Khoory et al. determined via PF4-Sepharose affinity chromatography that a coagulopathy in their patient was not due to a myeloma protein, but a circulating proteoglycan functioning as a cofactor for antithrombin III for which protamine sulfate led to correction of thrombin time [8]. Additionally, nearly equal inactivation of thrombin was achieved with standard beef lung heparin and the circulating heparin-like anticoagulant via in vitro measurement of plasma thrombin clot time [5]. In another case of IgG4 lambda multiple myeloma, an inhibitor most similar to heparan sulfate was isolated; the inhibitor acted as a cofactor for antithrombin III and was neutralized with protamine sulfate and platelet factor 4 [3]. Cases of both in vitro neutralization of the heparin-like anticoagulant with protamine sulfate, as well as in vivo neutralization, have been reported [5]. In one case, a 5 mg bolus of protamine sulfate was given to a de novo plasma cell neoplasm patient with epistaxis, mild hemoptysis, and bleeding surrounding a central venous catheter site, which led to improvement in thrombin clot time (TCT) for 24 hours, after which it became grossly abnormal (>120 sec) and required continuous infusion of protamine sulfate 2–7 mg/hour to correct the TCT [5]. While dosing remains unclear, in vitro studies of a patient with IgG-gamma myeloma showed escalating degrees of correction of thrombin time with increased doses of protamine [2]. Particularly, 10 mg/mL of protamine sulfate improved thrombin time from >600 s to 187 s, 50 mg/mL further improved thrombin time to 76 s, 100 mg/mL to 22 s, and 200 mg/mL to 21 s [2]. In another instance, plasmapheresis led to temporary resolution of bleeding in a patient with plasma cell neoplasia and myeloma kidney [9]. Plasmapheresis was not attempted in our patient given the high risk of bleeding associated with severe coagulopathy. DDAVP was attempted in our case, which did not lead to any improvement in coagulopathy. Alike our patient, Llamas et al. also reported a patient with plasma cell neoplasia that had non-life-threatening bleeding from a biopsy site, which required no intervention [10]. In a more recent analysis by Martínez-Martínez et al., liquid fast chromatography revealed an immunoglobulin-mediated disruption in the physiologic function of the thrombin-antithrombin complex, with a similar activation pattern to that of heparin [11]. Their patient was documented as having an IgG-gamma myeloma, and ours an IgG-kappa myeloma. More translational data are needed regarding paraprotein activation of the heparin-binding domain of antithrombin, as well as prevalence in other subtypes of myeloma. Acquisition of these data would have the potential to guide which patients are most appropriate to receive protamine in instances of bleeding relating to HLACs in multiple myeloma.

Our case, as well as other previously reported occurrences, illustrates that continuous protamine sulfate could assume the role of a primary treatment modality in patients with bleeding diathesis and plasma cell neoplasia with a suspected circulating heparin-like anticoagulant. Previous reports note that the circulating inhibitor produced in plasma cell neoplasia acts as a cofactor for antithrombin III and thus the pathophysiology also provides a rationale for protamine sulfate. It is possible that acquisition of an inhibitor is underdiagnosed in plasma cell neoplasia that causes primarily mucosal bleeding, which can be severe. This inhibitor in plasma cell neoplasia has been closely related to heparan sulfate in previous analyses, and further study could reveal if this molecule is made in homeostatic plasma cells at a possibly difficult to detect capacity. Other recent laboratory data demonstrate that the paraproteins have the ability to activate the antithrombin heparin-binding site, distinct from other molecules that may interact with the site, but not activate it. This emphasizes the need for further translational research to identify the origin of the HLAC, such that we can properly identify those patients that could benefit from protamine in cases of bleeding.
